# Effect of Trait Heritability, Training Population Size and Marker Density on Genomic Prediction Accuracy Estimation in 22 bi-parental Tropical Maize Populations

**DOI:** 10.3389/fpls.2017.01916

**Published:** 2017-11-08

**Authors:** Ao Zhang, Hongwu Wang, Yoseph Beyene, Kassa Semagn, Yubo Liu, Shiliang Cao, Zhenhai Cui, Yanye Ruan, Juan Burgueño, Felix San Vicente, Michael Olsen, Boddupalli M. Prasanna, José Crossa, Haiqiu Yu, Xuecai Zhang

**Affiliations:** ^1^College of Agronomy, Shenyang Agricultural University, Shenyang, China; ^2^International Maize and Wheat Improvement Center (CIMMYT), Texcoco, Mexico; ^3^National Engineering Laboratory for Crop Molecular Breeding, Institute of Crop Science, Chinese Academy of Agricultural Sciences, Beijing, China; ^4^International Maize and Wheat Improvement Center (CIMMYT), Nairobi, Kenya; ^5^Maize Research Institute, Heilongjiang Academy of Agricultural Sciences, Harbin, China

**Keywords:** maize, genomic selection, trait heritability, training population size, marker density

## Abstract

Genomic selection is being used increasingly in plant breeding to accelerate genetic gain per unit time. One of the most important applications of genomic selection in maize breeding is to predict and select the best un-phenotyped lines in bi-parental populations based on genomic estimated breeding values. In the present study, 22 bi-parental tropical maize populations genotyped with low density SNPs were used to evaluate the genomic prediction accuracy (*r*_*MG*_) of the six trait-environment combinations under various levels of training population size (TPS) and marker density (MD), and assess the effect of trait heritability (*h*^*2*^), TPS and MD on *r*_*MG*_ estimation. Our results showed that: (1) moderate *r*_*MG*_ values were obtained for different trait-environment combinations, when 50% of the total genotypes was used as training population and ~200 SNPs were used for prediction; (2) *r*_*MG*_ increased with an increase in *h*^*2*^, TPS and MD, both correlation and variance analyses showed that *h*^*2*^ is the most important factor and MD is the least important factor on *r*_*MG*_ estimation for most of the trait-environment combinations; (3) predictions between pairwise half-sib populations showed that the *r*_*MG*_ values for all the six trait-environment combinations were centered around zero, 49% predictions had *r*_*MG*_ values above zero; (4) the trend observed in *r*_*MG*_ differed with the trend observed in *r*_*MG*_/*h*, and *h* is the square root of heritability of the predicted trait, it indicated that both *r*_*MG*_ and *r*_*MG*_/*h* values should be presented in GS study to show the accuracy of genomic selection and the relative accuracy of genomic selection compared with phenotypic selection, respectively. This study provides useful information to maize breeders to design genomic selection workflow in their breeding programs.

## Introduction

Genomic selection (GS) is being used increasingly in plant breeding to accelerate genetic gain (Crossa et al., 2010; Zhang et al., 2015; Roorkiwal et al., 2016; Edriss et al., 2017). In GS, a training population is used for estimating the effects of markers based on prior phenotypic and marker data. The marker effects estimated from the training population are then used to predict the genomic estimated breeding value (GEBV) of individuals in the prediction population, which have been genotyped but not phenotyped (Meuwissen et al., 2001). Breeders then select individuals from the prediction population based on the GEBV to advance to the next cycle (generation) to shorten the length of the selection cycle and increase the genetic gain per unit time (Heffner et al., 2009; Lorenz et al., 2011; Guo et al., 2012; Newell and Jannink, 2014).

In plant breeding, several studies have implemented GS to develop improved germplasm, evaluate the genetic gain of GS (Poland et al., 2012; Battenfield et al., 2016; Marulanda et al., 2016), and compare the genetic gains from GS with marker assisted selection or pedigree selection methods (Asoro, 2013; Combs and Bernardo, 2013; Beyene et al., 2015; Rutkoski et al., 2015; Zhang et al., 2017). Using recombinant inbred lines derived from a cross between B73 and Mo17, Massman et al. (2013) showed that GS produced from 14 to 50% higher genetic gains for stover and grain yield than marker assisted recurrent selection for several traits in a maize biparental population. This result was then verified in tropical maize (Beyene et al., 2015; Vivek et al., 2017). Beyene et al. (2015) found that the average genetic gain per year across eight tropical maize populations of GS was three times higher than that of pedigree based phenotypic selection in drought stress conditions. Vivek et al. (2017) pointed that two cycles of GS produced 4–43% higher grain yield than two cycles of pedigree-based phenotypic selection in two bi-parental populations. In a multi-parental tropical maize population, Zhang et al. (2017) reported that the realized genetic gain of GS per year was 1.2%, when the total breeding time was estimated from making the initial cross to harvesting the last selection cycle. The average genetic gain per selection cycle was 2.8%, when only the selection cycles were considered as the total breeding time.

Prediction accuracy (*r*_*MG*_), defined as the correlation between the true breeding value and the GEBV, is used to evaluate the effectiveness of genomic selection, *r*_*MG*_ value must be high enough for GS to be time and cost effective (Combs and Bernardo, 2013). Highly variable levels of *r*_*MG*_ values have been reported in plants depending on prediction models, breeding schemes, training population size, the relationship between the training and the prediction populations, trait complexities, marker densities, and genotyping platforms (Jia and Jannink, 2012; Zhao et al., 2012; Crossa et al., 2013; Lian et al., 2014; Spindel et al., 2015; Battenfield et al., 2016; Bernardo, 2016). Previous studies showed that the *r*_*MG*_ value is increased as the increase of trait heritability, size of training population, and marker density. The *r*_*MG*_ value also could be improved, when the relationship between the training population and the prediction population is close (Sonah et al., 2015). Modeling genotyping by environment interactions and incorporating known marker-trait associations into prediction model is also beneficial increasing the *r*_*MG*_ value (Crossa et al., 2014; Zhang et al., 2015; Bian and Holland, 2017; Cao et al., 2017). Lian et al. (2014) showed that the *r*_*MG*_ is difficult to be predicted in advance, but *r*^*2*^(*Nh*^2^)^1/2^ had a strong association with the prediction accuracy value, where *r*^*2*^ refers to the linkage disequilibrium between a marker and a quantitative trait locus, *N* is the training population size, and *h*^*2*^ is the trait heritability. Further GS studies are still required to understand the factors that affect the prediction accuracy, and how to maximum the prediction accuracy to streamline GS schemes in a breeding program.

One of the most important applications of GS in maize breeding is to predict and identify the best untested lines from bi-parental populations, when the training and prediction populations are derived from the same cross. Moderate-to-high *r*_*MG*_ values have been reported in biparental populations due to the close relationship between the training and prediction populations, and the maximum linkage disequilibrium between a marker and a quantitative trait locus (Zhang et al., 2015). In this study, 22 bi-parental tropical maize populations including 4,120 segregating lines were phenotyped with six trait-environment combinations and each lines were genotyped with 162 to 283 SNPs (single nucleotide polymorphisms) (Semagn et al., 2014; Ertiro et al., 2015). The main objectives of this study were to: (1) evaluate the *r*_*MG*_ value of the six trait-environment combinations in 22 bi-parental tropical maize populations; (2) assess the effect of trait heritability, training population size (TPS) and marker density (MD) on *r*_*MG*_ estimation in bi-parental populations; (3) identify the most important factor affecting *r*_*MG*_ estimation and provide useful information to breeders for implementing GS in their breeding programs; and (4) assess the predictions accuracy between pairwise half-sib populations.

## Materials and methods

### Plant materials and phenotyping

This study comprised a total of 4,120 lines derived from 22 bi-parental populations. Sixteen of the 22 bi-parental populations were developed in 2009 as part of the Water Efficiency Maize for Africa project, and the other six bi-parental populations were developed as part of the Drought Tolerant Maize for Africa project in 2008. Details on population development and phenotyping were described in previous studies (Beyene et al., 2015; Zhang et al., 2015; Wallace et al., 2016). In brief, all the populations were derived from crosses between CIMMYT drought-tolerant donors and CIMMYT inbred lines currently in commercial use in eastern and southern Africa. The F_1_ crosses formed with two inbred line parents were advanced to the BC_1_F_2:3_, F_2:3_, or F_7:8_ generations for each population (details showed in the previous studies). Testcross hybrids of each population were generated by crossing the individual family with a single-cross tester from a complementary heterotic group. The testcrosses along commercial checks were planted under four well-watered (WW) environments and three to four water-stressed (WS) environments in Kenya, Zambia, and Zimbabwe during 2010 and 2011 (Semagn et al., 2013). The WW trials were planted during the rainy season, and supplemental irrigation was provided as needed. The WS trials were planted during the dry (rain-free) season by withdrawing irrigation starting from 2 weeks before flowering through harvest.

Parameter information of each population including pedigree code, tester, number of families in each population, number of polymorphic SNPs in each bi-parental population, mean, and standard deviation of all trait-environment combinations is summarized in Table 1. In total, 28 inbred lines were used as the parents to form the 22 bi-parental populations. Six testers were used in making testcrosses, of which 10 populations share tester T1, 6 populations share T2, 3 populations share T3, and the remaining 3 populations were testcrossed with T4, T5, or T6 (Table 1). The number of polymorphic SNPs in each population ranged from 162 to 283, with a mean of 206.

**Table 1:** Summary of 22 bi-parental populations used in the present study, including pedigree code, tester, number of families (N^a^) and number of polymorphic SNPs (N^b^) of all populations, mean and standard deviation of all target trait-environment combinations (GY, grain yield; AD, anthesis date; PH, plant height; WW, well-watered environments; WS, water-stressed environments).

**Pop**	**Pedigree**	**Tester**	**N^a^**	**N^b^**	**Mean** ± **Standard Deviation**					
**No**.	**code**				**GY_WW**	**GY_WS**	**AD_WW**	**AD_WS**	**PH_WW**	**PH_WS**
1	P1 × P2	T1	165	201	6.98 ± 1.91	2.34 ± 1.48	61.72 ± 8.28	86.78 ± 10.64	215.02 ± 37.06	151.68 ± 49.85
2	P2 × P3	T1	162	188	7.03 ± 2.12	2.69 ± 1.61	62.39 ± 8.57	84.48 ± 11.67	218.53 ± 37.91	152.50 ± 36.14
3	P2 × P4	T1	126	183	7.57 ± 2.64	2.34 ± 1.51	62.66 ± 8.55	86.3 ± 11.88	214.9 ± 39.08	160.57 ± 31.51
4	P2 × P5	T1	163	209	7.62 ± 2.32	2.76 ± 1.48	62.46 ± 8.87	82.17 ± 10.57	215.27 ± 37.9	156.78 ± 34.66
5	P4 × P1	T1	183	208	6.27 ± 2.37	2.81 ± 1.67	64.97 ± 8.25	83.88 ± 9.77	199.38 ± 46.76	155.99 ± 36.42
6	P6 × P7	T1	173	195	6.32 ± 2.15	2.70 ± 1.88	60.32 ± 7.43	74.18 ± 10.83	180.97 ± 34.53	160.08 ± 27.26
7	P8 × P9	T1	181	212	6.67 ± 1.98	2.55 ± 1.69	61.37 ± 6.75	75.22 ± 10.08	192.42 ± 38.06	169.55 ± 22.55
8	P8 × P7	T1	184	212	6.67 ± 1.92	2.59 ± 1.28	60.43 ± 5.15	81.88 ± 11.25	174.38 ± 40.46	139.08 ± 35.87
9	P10 × P11	T1	184	211	7.06 ± 1.91	1.75 ± 1.50	64.26 ± 8.33	89.67 ± 12.57	216.63 ± 44.08	154.39 ± 24.57
10	P12 × P13	T1	174	194	7.31 ± 2.23	2.44 ± 1.62	65.20 ± 8.17	88.47 ± 11.04	220.70 ± 45.49	166.41 ± 29.92
11	P17 × P18	T2	184	185	5.45 ± 1.91	1.99 ± 1.33	63.55 ± 8.10	86.28 ± 17.74	243.99 ± 35.67	177.80 ± 23.01
12	P18 × P19	T2	160	162	6.66 ± 2.84	2.38 ± 1.23	66.04 ± 9.09	84.84 ± 12.85	243.56 ± 38.78	180.78 ± 22.17
13	P19 × P15	T2	178	176	6.38 ± 2.73	1.90 ± 1.05	65.76 ± 8.86	84.83 ± 94.46	242.57 ± 46.75	172.52 ± 24.19
14	P20 × P17	T2	173	166	6.95 ± 2.86	2.03 ± 1.16	64.14 ± 8.77	87.85 ± 12.61	232.14 ± 42.01	166.06 ± 36.08
15	P21 × P22	T2	176	172	6.82 ± 2.87	2.04 ± 1.06	63.08 ± 8.20	85.00 ± 11.33	219.31 ± 33.64	156.47 ± 23.74
16	P22 × P23	T2	155	184	6.63 ± 2.61	1.75 ± 0.99	64.01 ± 8.43	85.22 ± 11.32	233.82 ± 41.48	158.36 ± 28.42
17	P19 × P15	T3	164	255	9.11 ± 0.91	5.55 ± 0.80	100.81 ± 1.56	98.97 ± 1.25	223.02 ± 7.81	192.59 ± 6.87
18	P19 × P26	T3	278	283	9.91 ± 1.31	3.66 ± 0.66	65.64 ± 0.95	79.93 ± 1.57	247.07 ± 10.13	192.29 ± 8.19
19	P19 × P27	T3	216	217	8.37 ± 1.26	4.64 ± 0.94	62.03 ± 1.59	85.59 ± 1.43	251.26 ± 10.49	223.70 ± 8.93
20	P24 × P25	T4	247	199	10.00 ± 1.31	4.88 ± 0.55	62.32 ± 1.04	85.12 ± 1.23	233.92 ± 10.11	211.86 ± 7.68
21	P28 × P29	T5	249	238	2.54 ± 0.48	1.54 ± 0.28	54.38 ± 0.76	64.79 ± 1.38	155.07 ± 9.90	126.86 ± 6.66
22	P30 × P31	T6	245	271	5.38 ± 0.72	1.56 ± 0.33	55.46 ± 0.91	70.82 ± 0.98	232.45 ± 10.94	169.63 ± 7.40

All the testcross hybrids in each population were evaluated up to 17 different traits, but only grain yield (GY), anthesis date (AD), and plant height (PH) were selected as the main target traits in the present study. GY was measured as dry shelled grain yield at 12.5% moisture content. AD was measured as the number of days from planting to when 50% of the plants had shed pollen. PH was measured as the distance from the base of the plant to the height of the first tassel branch. In total, six trait-environment combinations (that is, GY_WW, AD_WW, PH_WW, GY_WS, AD_WS and PH_WS) were considered, GY_WW and GY_WS were treated as complex traits, and the other four trait-environment combinations were treated as less complex traits in the present study.

### Phenotypic data analysis and heritability estimation

The experimental design in each environment was an α-lattice incomplete block design with two replications per location. At all locations, entries were planted in one-row plot with 5 m long, 0.75 m between rows and 0.25 m between hills. For each population, combined trial analyses were performed within WW and WS environments, respectively.

MEATA-R software (http://hdl.handle.net/11529/10201) was used to conduct multi-location trial analysis using a mixed linear model with all factors set as random effects. Best linear unbiased prediction (BLUP) value of genotypes, variance components and broad-sense heritability were obtained. Broad-sense heritability of the target trait was calculated as the ratio of total genetic to total phenotypic variance. In multi-location trial analysis, broad-sense heritability was calculated as

$$h^{2}=\frac{\sigma_{g}^{2}}{\sigma_{g}^{2}+\frac{\sigma_{ge}^{2}}{e}+\frac{\sigma_{e}^{2}}{er}}$$

Where $\sigma_{g}^{2},\;\sigma_{ge}^{2},and\;\sigma_{e}^{2}$ are the genotypic, genotype-by-environment interaction, and error variance components, respectively, and e and r are the number of environments and of replicates within each environment included in the corresponding analysis, respectively. According to the *h*^*2*^ value of each trait-environment combination, the 22 populations were sorted in an ascending order and divided into 4 subgroups for estimating the relationship between *h*^*2*^ and genomic prediction accuracy. Subgroup 1 (lowest heritability subgroup) had six populations, and the other three subgroups each had five populations.

### Genotyping

The 16 populations developed for the Water Efficiency Maize for Africa project were genotyped by the Monsanto Company using a TaqMan assay (http://www.appliedbiosystems.com), while the 6 populations developed for the Drought Tolerant Maize for Africa project were genotyped at LGC genomics (http://www.lgcgenomics.com/genotyping/kasp-genotyping-chemistry) using a KASP assay (Semagn et al., 2014). For each family in each population, equal amount of leaf tissue from 15 plants were bulked for DNA extraction and genotyping. For each segregating SNP, a χ2 goodness-of-fit analysis was performed to test for deviation from the expected segregation ratio. Only SNPs passed the segregation distortion tested were used for further analyses.

### Genome-wide prediction

Genome-wide prediction was performed using the rrBLUP package for R version 3.2.5 (Endelman, 2011). In order to test the effects of TPS and MD on genomic prediction accuracy, four levels of TPS (i.e., 30, 50, 70, and 90) and three levels of MD (i.e., 50 SNPs, 100 SNPs, and all SNPs) were considered to evaluate the prediction accuracy of each trait-environment combination within each bi-parental population. Different with the 5-fold cross-validation method reported previously (Zhang et al., 2015; Cao et al., 2017), fixed number of lines were randomly sampled 100 times from each bi-parental population to develop the prediction model. Approximate 16, 28, 39, and 50% of the entire population were selected to build the training population, when the TPS was 30, 50, 70, and 90, respectively. All the predictions were replicated for 100 times, the average value of the Pearson correlations between the phenotype and the genomic estimated breeding values was defined as the genomic prediction accuracy (*r*_*MG*_). For each trait-environment combination, value of *r*_*MG*_/*h* was calculated to compare the breeding efficiency between phenotypic selection and genomic selection, where *h* was the square root of the heritability of the target trait-environment combination, and the accuracy of phenotypic selection was measured by *h*. In total, we did prediction on 1,584 scenarios, when all the possible recombination were considered among 22 populations, 6 trait-environment combinations, 4 levels of TPS, and 3 levels of MD.

Predictions between pairwise half-sib populations were performed, when two populations shared one common parental line, and one population was used as training population to predict the other population. In the prediction population, the Pearson correlations value between the phenotype and the genomic estimated breeding values was defined as *r*_*MG*_. In total, 204 predictions were made on the six trait-environment combinations between all the 34 pairwise half-sib populations, only the common markers shared between the pairwise half-sib populations were used for prediction. Due to lack of common markers, predictions across different genotyping platforms (TaqMan assay and KASP assay) were not conducted.

### Correlation and variance analyses between *r_*MG*_* and three factors

Correlation and variance analyses were performed across all the 22 bi-parental populations to see the importance of the three factors (i.e., *h*^*2*^, TPS, and MD) on affecting *r*_*MG*_, correlation coefficient between *r*_*MG*_ and three factors across all the 22 bi-parental populations were estimated in R version 3.2.5 (R Development Core Team, 2013) on all the six trait-environment combinations.

In the variance analysis, *r*_*MG*_ was set as response variable, and *h*^*2*^, TPS and MD were set as predictor variables, where the importance of the three factors will be assessed on all the six trait-environment combinations across all the 22 bi-parental populations.

## Results

### Phenotypes and heritability

For all the 22 bi-parental populations, the phenotypic mean and standard deviation of the six trait-environment combinations were showed in Table 1. Phenotypic mean of GY and PH were consistently higher under WW condition than under WS condition in all the populations. Phenotypic mean of GY ranged from 2.54 to 9.91 t/ha, with an average value of 6.99 t/ha under WW condition, while the mean of GY ranged from 1.54 to 4.88 t/ha, with an average value of 2.68 t/ha under WS condition. For PH_WW, the phenotypic mean ranged from 155.07 to 251.26 cm, and have an average value of 218.47 cm across all the populations. For PH_WS, the phenotypic mean ranged from 126.86 to 223.7 cm, and have an average value of 168 cm across all the populations. Phenotypic mean of AD was consistently lower under WW condition than under WS condition in all the populations. Phenotypic mean of AD ranged from 54.38 to 100.81 days, with an average value of 64.23 days under WW condition, while the mean of AD ranged from 64.79 to 98.97 days, with an average value of 83.29 days under WS condition across all the populations. The phenotypic mean distribution of all the six trait-environment combinations was either normal or approximately normal for all the 22 bi-parental populations (data not shown). Standard deviation of GY were consistently greater under WW condition than under WS condition in all the populations, while standard deviation of the less complex traits, i.e., AD and PH, were consistently smaller under WW condition than under WS condition.

**Figure 1:**
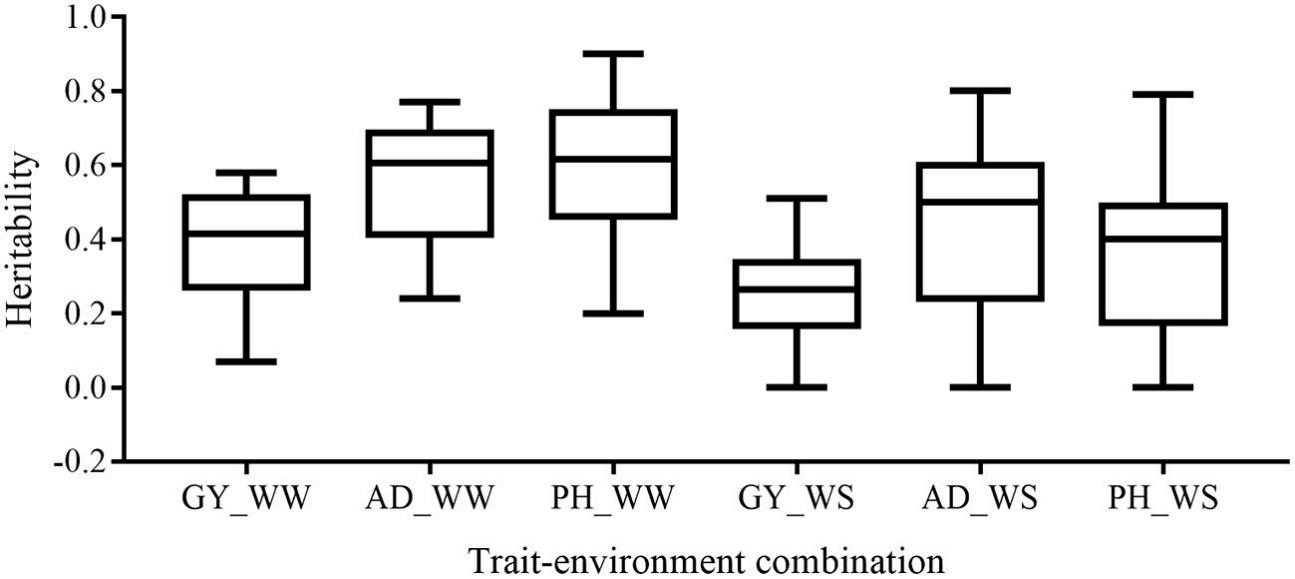
Distribution of heritability of all the target trait-environment combinations across all the populations.

Broad-sense heritability of the six trait-environment combinations in all the 22 bi-parental populations was shown in Figure 1 and Table S1. *h*^*2*^ of the complex trait (GY) were consistently lower than those of less complex traits (AD and PH) under both WW and WS conditions. For the same trait, *h*^*2*^ under WW conditions were consistently higher than under WS conditions in almost all the populations. Under WW condition, *h*^*2*^ across populations had a mean value of 0.38, 0.55 and 0.59 for GY, AD, and PH, respectively. Under WS condition, *h*^*2*^ across populations had a mean value of 0.27, 0.47, and 0.37 for GY, AD, and PH, respectively.

### Prediction accuracy mean and effect of heritability on prediction accuracy estimation

**Figure 2:**
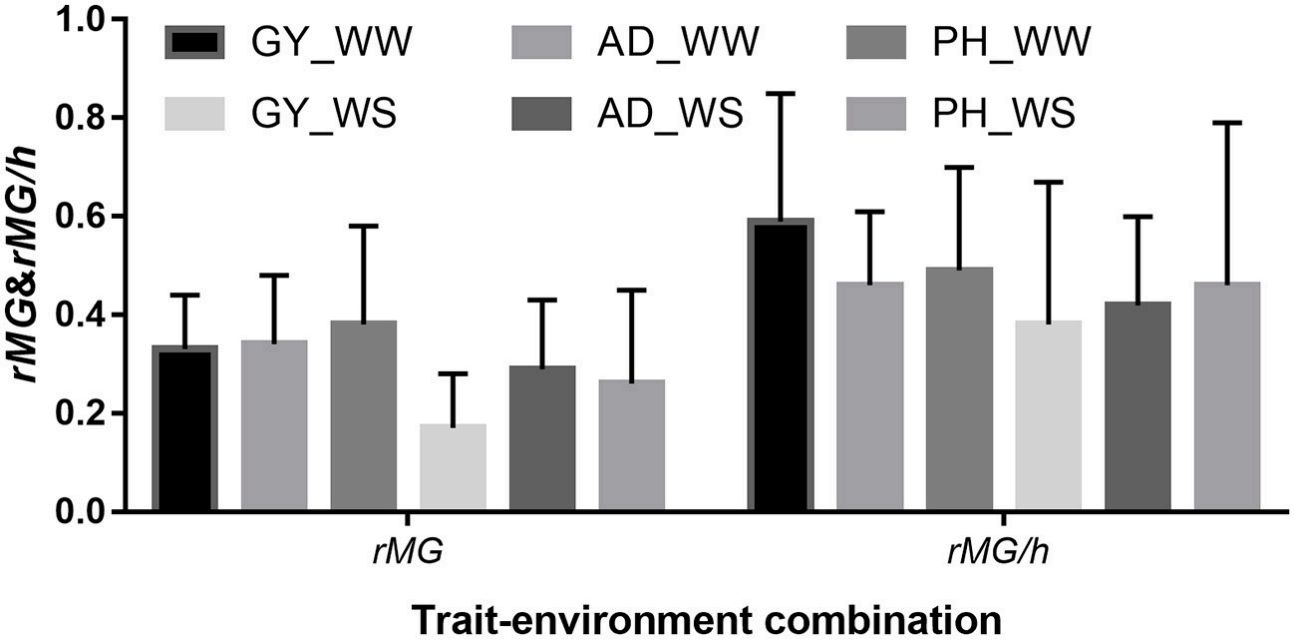
Mean and standard deviation (SD) of *r*_*MG*_ and *r*_*MG*_/*h* of all the target traits across all the 22 bi-parental populations. Values of *r*_*MG*_ in each population were estimated, when training population size equaled to 90 and all the SNPs were used for prediction.

**Figure 3:**
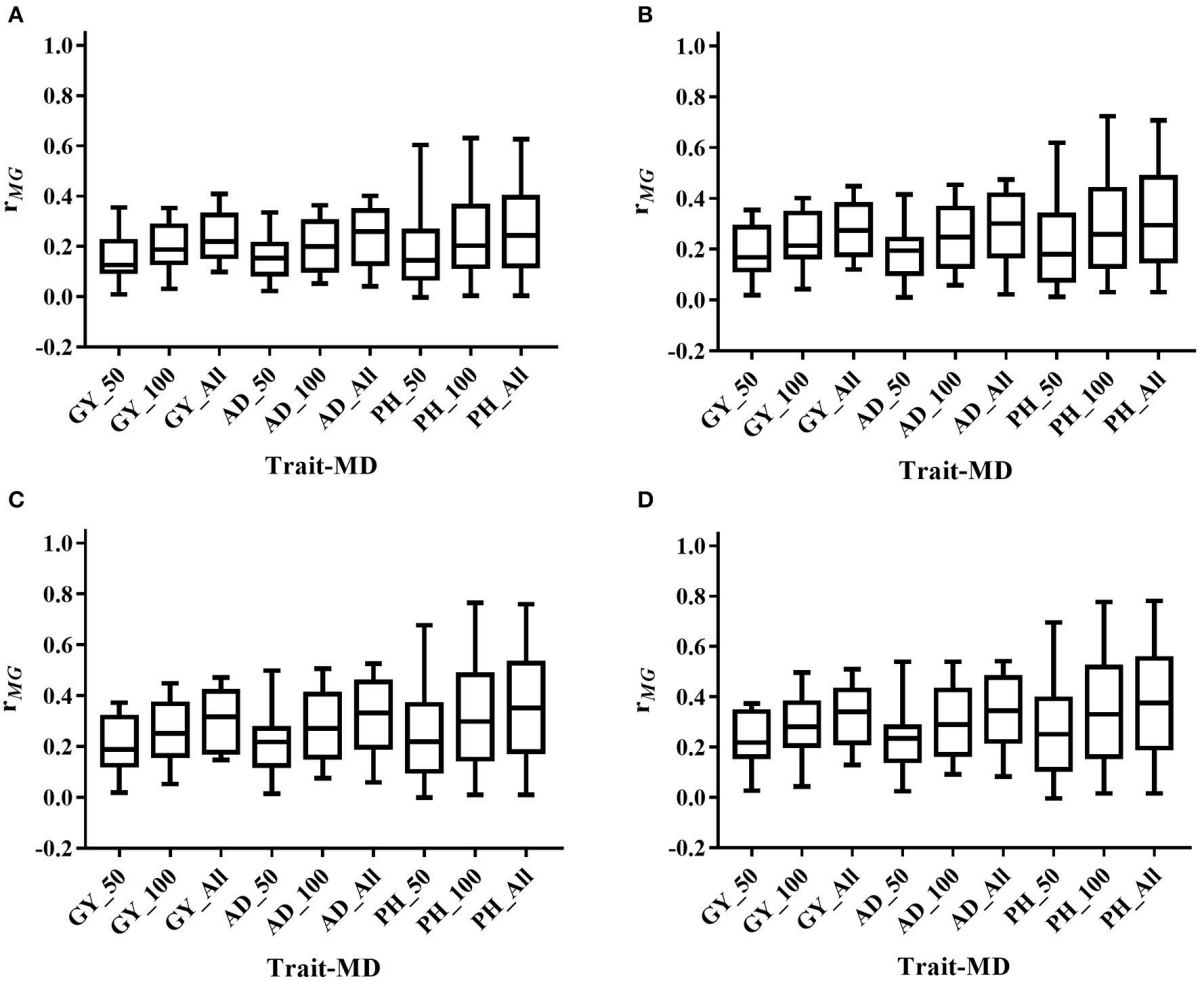
Distribution of *r*_*MG*_ across all the populations for all the target traits evaluated under WW condition under all the possible training population size (TPS) and marker density (MD) combinations. **(A)** TPS = 30; **(B)** TPS = 50; **(C)** TPS = 70; **(D)** TPS = 90. Three levels of MD, i.e., 50 SNPs, 100 SNPs and All SNPs, were used for prediction.

**Figure 4:**
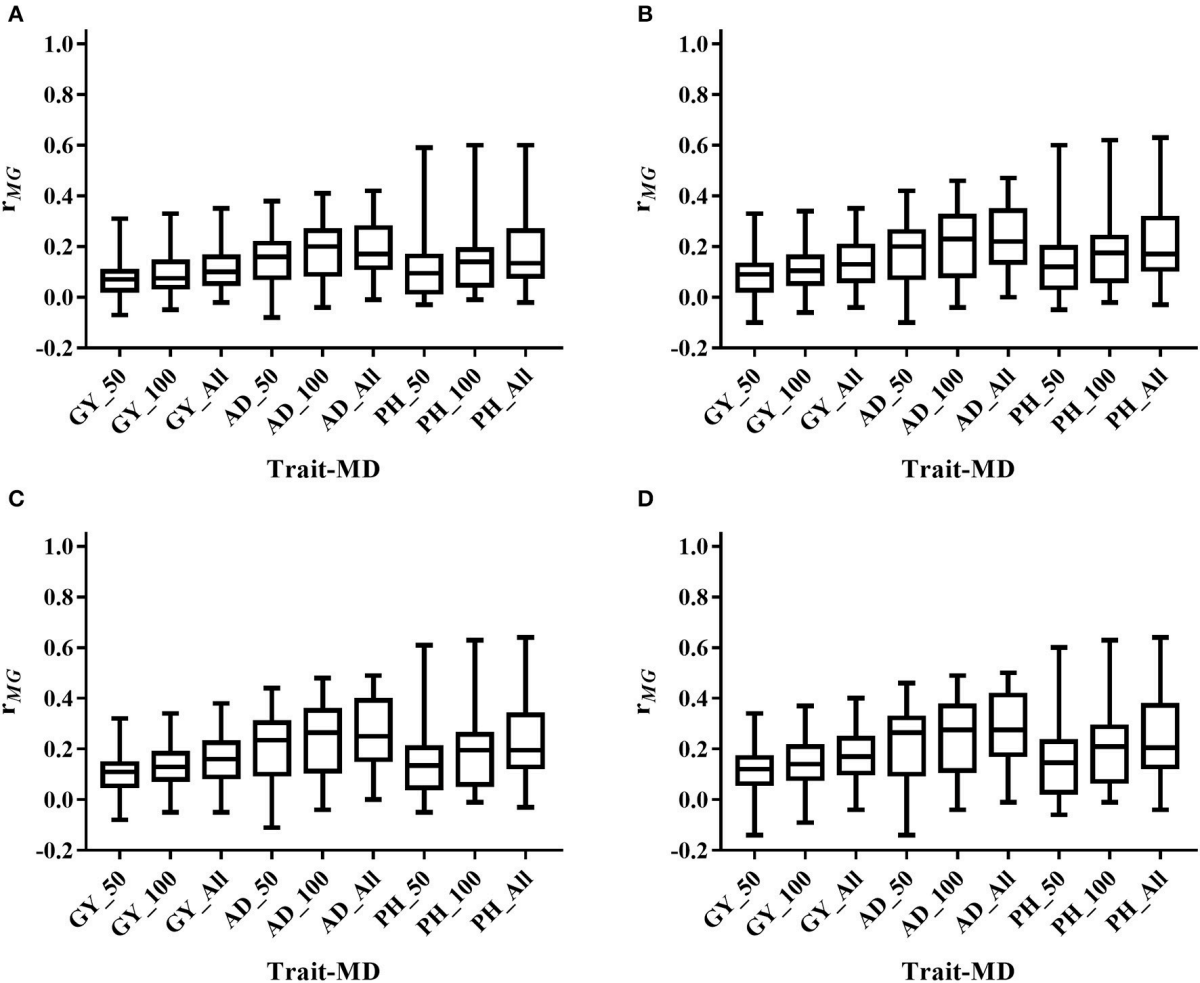
Distribution of *r*_*MG*_ across all the populations for all the target traits evaluated under WS condition under all the possible training population size (TPS) and marker density (MD) combinations. **(A)** TPS = 30; **(B)** TPS = 50; **(C)** TPS = 70; **(D)** TPS = 90. Three levels of MD, i.e., 50 SNPs, 100 SNPs and All SNPs, were used for prediction.

The mean, range and standard deviation of *r*_*MG*_ differed among the six trait-environment combinations (Figure 2, Table S2). In general, trait-environment combination with higher *h*^*2*^ had a higher *r*_*MG*_. Mean of *r*_*MG*_ of complex trait (GY) was consistently lower than those of less complex traits (AD and PH) under both WW and WS conditions. For the same trait, *r*_*MG*_ mean under WW condition was consistently higher than under WS condition. The *r*_*MG*_ mean was lowest for GY_WS and highest for PH_WW. For example, when TPS equaled to 90 and all the SNPs were used for prediction, *r*_*MG*_ across populations under WW condition had a mean value of 0.33, 0.34, and 0.38 for GY, AD, and PH, respectively (Figure 2, Table S2). The *r*_*MG*_ across populations under WS condition had a mean value of 0.18, 0.28, and 0.25 for GY, AD, and PH, respectively (Figure 2, Table S2). For the same trait, the standard deviation of *r*_*MG*_ under WW condition was similar with that under WS condition. Similar trends were observed, when other TPS and MD combinations were used for prediction (Figures 3, 4).

**Figure 5:**
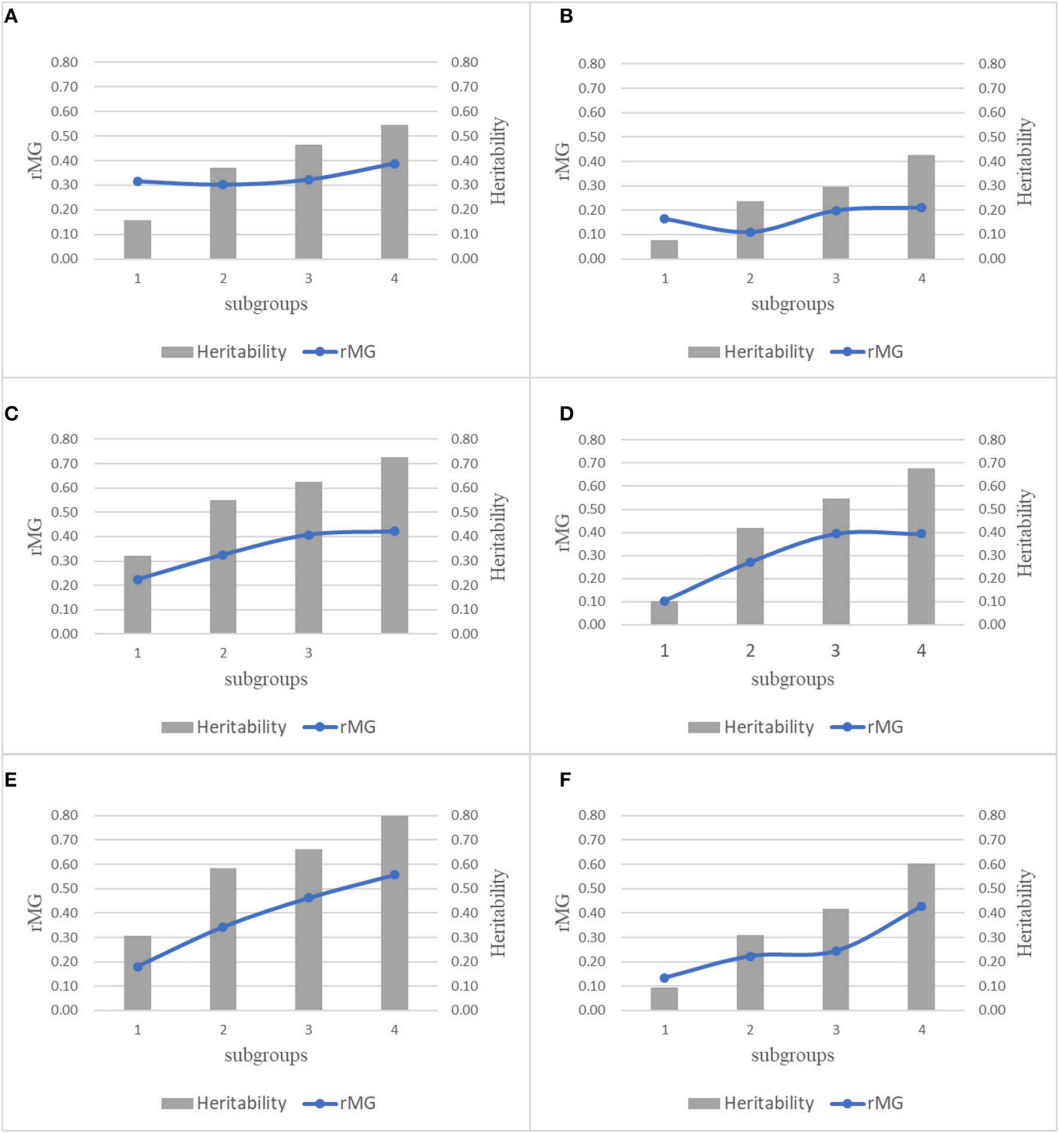
Combination plot of *r*_*MG*_ and *h*^*2*^ of all the 6 trait-environment combinations, all the 22 populations were divided into 4 subgroups sorted by the *h*^*2*^ of target traits from low to high, mean *r*_*MG*_ of each subgroup was estimated under combinations of TPS = 90 and MD = All SNPs. **(A)** GY_WW; **(B)** GY_WS; **(C)** AD_WW; **(D)** AD_WS; **(E)** PH_WW; **(F)** PH_WS.

For each trait-environment combination, the *r*_*MG*_ differed among populations, where populations with higher *h*^*2*^ had higher *r*_*MG*_ values (Figure 5). For all the six trait-environment combinations, *r*_*MG*_ mean estimated under combinations of TPS = 90 and MD = All SNPs were used as example and to show the correlations between *r*_*MG*_ and *h*^*2*^, where 22 populations were divided into 4 subgroups sorted by the *h*^*2*^ of each trait-environment combination from low to high. In general, the *r*_*MG*_ value increased with the increase of *h*^*2*^ for all the trait-environment combinations, but the trends differed among trait-environment combination. For the complex traits (GY_WW and GY_WS), slight increase was observed with the increase of *h*^*2*^ among subgroups. Slight decrease was observed from subgroup 1 to subgroup 2, due to sampling variation/error in both *r*_*MG*_ and *h*^*2*^ in subgroup 1, it indicated that other factors, i.e., TPS and MD, are important for further improvement on *r*_*MG*_ estimation of GY. For less complex trait AD, the increase in *r*_*MG*_ began to plateau once a high *h*^*2*^ was reached (subgroups 3 and 4). For less complex trait PH, the *r*_*MG*_ continuously increased with the increase of *h*^*2*^ from subgroup 1 to subgroup 4, it indicated that further improvement on *r*_*MG*_ of PH could be obtained by increasing the *h*^*2*^.

The mean, range and standard deviation of *r*_*MG*_/*h* differed among the six trait-environment combinations (Figure 2). However, the trend observed in *r*_*MG*_/*h* different with the trend observed in *r*_*MG*_ for the different trait-environment combinations. The highest *r*_*MG*_/*h* mean was observed on complex trait GY under WW condition. In contrast, complex trait GY had the lowest *r*_*MG*_/*h* mean among the three traits evaluated under WS condition. For the same trait, *r*_*MG*_/*h* mean value under WW condition was consistently higher than under WS condition. When TPS equaled to 90 and all the SNPs were used for prediction, *r*_*MG*_/*h* value across populations under WW condition had a mean of 0.59, 0.46, and 0.49 for GY, AD, and PH, respectively. Under WS condition, *r*_*MG*_/*h* value across populations had a mean of 0.38, 0.42, and 0.46 for GY, AD, and PH, respectively.

### Effect of training population size on prediction accuracy estimation

Prediction accuracy increased as the TPS increased for all the trait-environment combinations, when the MD was constant (Figures 3, 4, Table S2). When all the SNPs were used for prediction, the *r*_*MG*_ of GY_WW was 0.24 with TPS = 30, 0.28 with TPS = 50, 0.31 with TPS = 70, and 0.33 with TPS = 90. The *r*_*MG*_ mean of AD_WW was 0.24 with TPS = 30, 0.29 with TPS = 50, 0.32 with TPS = 70, and 0.34 with TPS = 90. The *r*_*MG*_ mean of PH_WW was 0.27 with TPS = 30, 0.32 with TPS = 50, 0.36 with TPS = 70, and 0.38 with TPS = 90. The similar trend was observed with MD = 50 and MD = 100. The *r*_*MG*_ mean of the target traits evaluated under WS condition also increased as the TPS increased, when the MD was constant.

### Effect of marker density on prediction accuracy estimation

Prediction accuracy increased as the MD increased for all the trait-environment combinations, when the TPS was constant (Figures 3, 4, Table S2). When TPS equaled to 90, the *r*_*MG*_ mean of GY_WW was 0.24 with MD = 50, 0.29 with MD = 100, and 0.33 with MD = all of markers. The *r*_*MG*_ mean of AD_WW was 0.24 with MD = 50, 0.31 with MD = 100, and 0.34 with MD = all of markers. The *r*_*MG*_ mean of PH_WW was 0.27 with MD = 50, 0.34 with MD = 100, and 0.38 with MD = all of markers. The similar trend was observed with TPS = 30, TPS = 50 and TPS = 70. The *r*_*MG*_ mean of the target traits evaluated under WS condition also increased as the MD increased, when the TPS was constant.

### Predictions between pairwise half-sib populations

**Figure 6:**
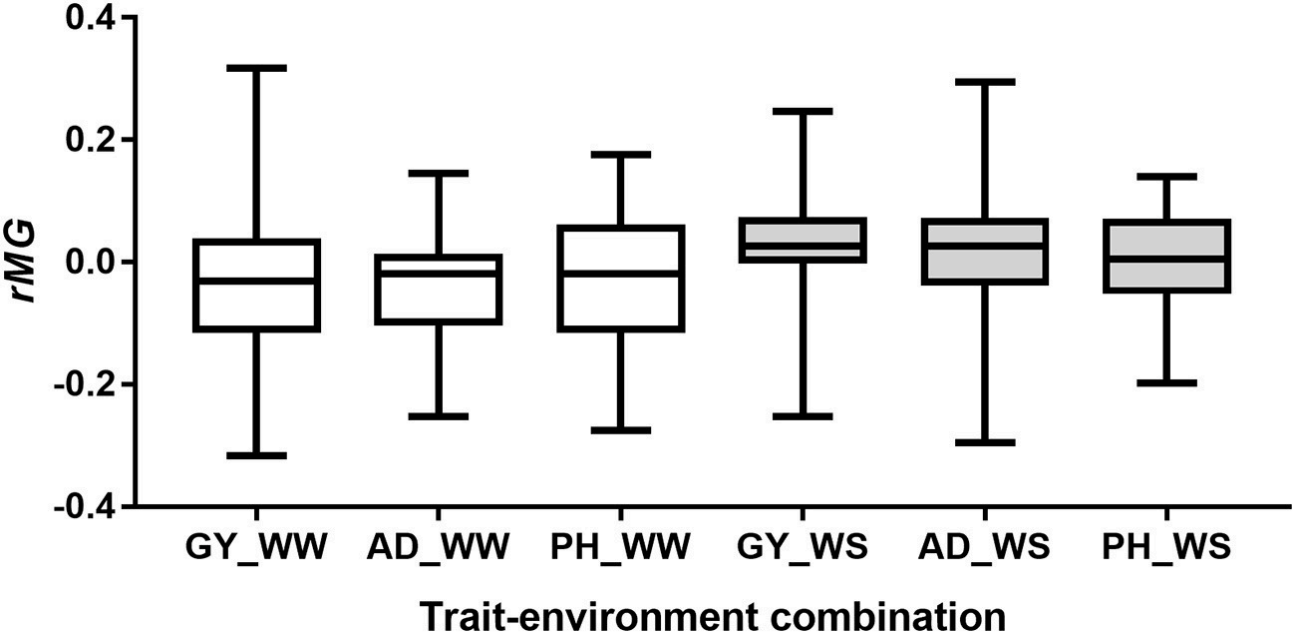
Distribution of *r*_*MG*_ values of predictions between the pairwise half-sib populations.

Number of markers used for predictions between the pairwise half-sib populations ranged from 44 to 100, with a mean of 71 (Table S3). The observed *r*_*MG*_ values between the pairwise half-sib populations were centered around zero for all the six trait-environment combinations (Figure 6). Out of the 204 predictions, 100 (49%) had *r*_*MG*_ values above zero. The maximum *r*_*MG*_ value of the target traits evaluated under WW condition was 0.32, 0.14, and 0.18 for GY, AD, and PH, respectively. While the maximum *r*_*MG*_ value of the target traits evaluated under WS condition was 0.25, 0.30, and 0.14 for GY, AD, and PH, respectively (Table S3).

### Association between prediction accuracy and all the three factors

Correlation analysis results between *r*_*MG*_ and individual factor (*h*^*2*^, TPS, and MD) were showed in Table 2, which differed among the different trait-environment combinations. The *r*_*MG*_ was most strongly associated with *h*^*2*^ for all the trait-environment combinations except for GY_WW. The second strongly associated factor was TPS for all the trait-environment combinations except for GY_WS. The *r*_*MG*_ was least associated with MD for all the trait-environment combinations except for GY_WS. Compared with the less complex traits (AD and PH), the correlation values between *r*_*MG*_ and *h*^*2*^ of the complex traits (GY) were lower, it indicated that all the three factors have to be considered simultaneously for further improvement on *r*_*MG*_ estimation for GY. For the less complex traits, *h*^*2*^ of the predicted trait was the most important factor for *r*_*MG*_ estimation improvement.

**Table 2:** Correlation between *r*_*MG*_ and the three factors (*h*^*2*^, TPS and MD) for all the trait-environment combinations.

**Trait**	***h*^2^**		**TPS**		**MD**	
	**Correlation**	**P value**	**Correlation**	**P value**	**Correlation**	**P value**
GY_WW	0.17	4.66E-03	0.29	1.49E-06	0.25	5.12E-05
GY_WS	0.49	2.24E-17	0.19	2.01E-03	0.24	7.57E-05
AD_WW	0.51	6.00E-19	0.26	1.37E-05	0.22	3.45E-04
AD_WS	0.72	3.91E-43	0.21	4.68E-04	0.16	8.28E-03
PH_WW	0.72	4.90E-43	0.19	2.05E-03	0.13	3.39E-02
PH_WS	0.75	4.93E-49	0.12	5.97E-02	0.9.0	1.45E-01

Variance analysis results between the *r*_*MG*_ and all three factors together were shown in Table 3. Total variance explained by the three factors were lower for complex traits than less complex traits. In the regression model, the total variance explained by the three factors was 17.69, 39.18, and 58.85% for traits of GY, AD, and PH evaluated under WW condition, while the total variance explained by the three factors was 32.51, 57.68, and 58.62% for GY, AD, and PH evaluated under WS condition. For the same trait, the percentage of total variance explained was higher or similar under WS condition than under WW condition. Among the three factors, *h*^*2*^ explained the greatest percentage of the total variance for all the trait-environment combinations except GY_WW, and MD explained the least percentage of the total variance for all the trait-environment combinations except GY_WS and PH_WW. Variance analysis results were consistent with the results observed in correlation analysis.

**Table 3:** Variance analysis between *r*_*MG*_ and the three factors (*h*^*2*^, TPS and MD) for all the trait-environment combinations.

**Trait**	**h2**		**TPS**		**MD**		**Residuals**
	**Variance explained (%)**	**P value**	**Variance explained (%)**	***P*-value**	**Variance explained (%)**	***P*-value**	**Variance explained (%)**
GY_WW	3.02	2.34E-03	8.58	1.14E-05	6.10	1.79E-05	82.31
GY_WS	24.04	8.09E-19	3.63	3.59E-03	4.84	2.42E-05	67.49
AD_WW	26.09	9.00E-22	7.18	2.40E-06	5.90	1.04E-06	60.82
AD_WS	51.56	1.54E-46	4.66	5.95E-06	1.46	3.15E-03	42.32
PH_WW	51.48	2.32E-47	3.67	6.36E-05	3.70	2.47E-06	41.15
PH_WS	56.32	4.79E-50	1.43	3.20E-02	0.87	2.06E-02	41.38

## Discussion

In maize breeding, one of the most promising applications of GS is to predict and select the best un-phenotyped lines in a bi-parental population, when a subset of this population has been phenotyped and genotyped as a training population, moderate-to high *r*_*MG*_ value for various traits could be obtained using different TPS and MD combinations (Crossa et al., 2014; Zhang et al., 2015). In the present study, 22 bi-parental tropical maize populations were used to assess the effect of *h*^*2*^, TPS, and MD on *r*_*MG*_ estimation. Results showed that *r*_*MG*_ value increase with an increase in *h*^*2*^, TPS, and MD. The correlation between *h*^*2*^ and *r*_*MG*_ was significant for all the trait-environment combinations. Trait-environment combinations with higher *h*^*2*^ had higher *r*_*MG*_values, and *r*_*MG*_ mean of less complex trait was consistently higher than that of complex trait under both conditions. For the same trait, *r*_*MG*_ mean under WW condition was consistently higher than that under WS condition, and populations with higher *h*^*2*^ of the target trait-environment combination also had higher *r*_*MG*_ values than that of populations with lower *h*^*2*^. Variance analysis also showed that *h*^*2*^ is the most important factors on *r*_*MG*_ estimation, and *h*^*2*^ explained the greatest percentage of the total variance for all the trait-environment combinations, only except for GY_WW. Our results agree with the previous studies that increase in *h*^*2*^ of the target trait results in an increase in *r*_*MG*_ (Combs and Bernardo, 2013; Lian et al., 2014). When the breeders design the GS pipeline in their breeding programs, they should consider that the heritability of the target traits in training population must be high to achieve good prediction accuracy values by increasing the number of locations and replications in phenotyping trials.

In both correlation and variance analyses, results showed that the *r*_*MG*_ was significantly associated with TPS for all the trait-environment combinations, only except for PH_WS. This result also agrees with the previous studies that increase in TPS results in an increase in *r*_*MG*_ (Zhang et al., 2015; Cao et al., 2017). Optimal TPS for running GS within a bi-parental population is also of interest of maize breeder. Cao et al. (2017) reported that relative high *r*_*MG*_ with the smallest standard error were observed when 50% of the total genotypes were used as a training population. Different with the 5-fold cross-validation method reported previously, fixed number of lines were randomly sampled 100 times to evaluate the *r*_*MG*_ value of different target trait-environment combinations in the present study. Our results also confirm that moderate-to high *r*_*MG*_ values of a wide range of target trait-environment combinations obtained, when half of the population is used to build the prediction model. However, the *r*_*MG*_ value could vary depending the genetic complexity of the target traits and the total population size used for sampling training population.

Among the three factors, the *r*_*MG*_ was least associated with MD for most of the trait-environment combinations, although the *r*_*MG*_ increases as the MD increases. In the correlation analysis, the correlation values between *r*_*MG*_ and MD of the complex traits were higher as compared with the less complex traits under both the WW and WS conditions, which indicated higher MD is still required to obtain good *r*_*MG*_ values for complex traits with relative low *h*^*2*^. The variance analysis showed that the MD explained greater variance in complex traits than in less complex traits, which indicated that MD plays a more important role on improving the *r*_*MG*_ value of the complex traits. Our results are consistent with the previous studies, good *r*_*MG*_ values could be obtained in bi-parental maize populations, when the *h*^*2*^ of the target trait is high and the genome is covered with sufficient markers, i.e., mean distance between markers is < 10–20 cM or around 150 markers evenly distributed the whole genome (Albrecht et al., 2011; Windhausen et al., 2012; Gorjanc et al., 2016). Higher MD is required to achieve good *r*_*MG*_ values in bi-parental populations for complex traits with relative low *h*^*2*^ and strong genotype by environment interaction (Lorenzana and Bernardo, 2009; Lian et al., 2014; Zhang et al., 2015). In the previous studies, either *r*_*MG*_ or *r*_*MG*_/*h* was used to assess the prediction ability (Crossa et al., 2014; Bernardo, 2016), the accuracy of GS is measured by *r*_*MG*_ (the average value of the Pearson correlations between the phenotype and the genomic estimated breeding values), and the accuracy of phenotypic selection is measured by *h* (the square root of heritability of the predicted trait). The relative accuracy of GS compared with phenotypic selection represents by the value of *r*_*MG*_/*h*. In this study, both *r*_*MG*_ and *r*_*MG*_/*h* values were presented. However, the trend observed in *r*_*MG*_ differed with the trend observed in *r*_*MG*_/*h* for the different trait-environment combinations. Under WW condition, GY had a lowest *r*_*MG*_ mean and a highest *r*_*MG*_/*h* mean among all the three traits, and it indicated that GS is more effective for complex trait improvement than phenotypic selection. In contrast, GY becomes the most ineffective trait for GS due to the lowest *r*_*MG*_ and *r*_*MG*_/*h* means among all the three traits evaluated under WS condition. Lowest *r*_*MG*_ and *r*_*MG*_/*h* of GY_WS are mainly caused by its relative low *h*^*2*^, which indicates the importance of improving the *h*^*2*^ of the predicted trait in training population.

Genomic prediction also could be improved by pooling multiple related bi-parental populations into the training sets or using multi-parental populations (Schulz-Streeckab et al., 2012; Zhang et al., 2015). However, only predictions between pairwise half-sib populations were performed in the present study, pooling multiple related populations as training population to predict the other related or un-related populations were not applied. Because the low-density markers were used to genotype each bi-parental population, and only few number of markers shared among the multiple populations. Predictions between pairwise half-sib populations showed that the *r*_*MG*_ values for all the six trait-environment combinations were centered around zero, it indicated that the predictions between pairwise half-sib populations does not work well, especially with less number of shared markers among populations. Good predictions by pooling multiple related populations as training population require genotyping all the populations with high-density markers to have enough number of common markers among all the populations.

High-throughput and cost-effective genotyping platforms are required to implement GS routinely in the breeding programs. Recently advances in bar-coded multiplexed sequencing technologies, such as genotyping-by sequencing (GBS), provide the capacity to genotype a large number of breeding lines at low costs (Elshire et al., 2011; Wu et al., 2016). GBS multiplexed 96 to 384 samples into one sequencing lane has a similar cost with the low density SNPs obtained from the single-plex arrays. GBS becomes a competitive alternative for increasing the number of markers many folds economically for running GS on crop improvement, several previous studies showed that GBS could improve genomic prediction accuracy compared with the low density SNPs at similar costs (Crossa et al., 2013; Zhang et al., 2015). In this study, our results showed that high-density genotyping platforms are required, when the predictions are applied on complex traits with low heritability or by pooling multiple related bi-parental populations as training population. rAmpSeq (repeat Amplification Sequencing; (Buckler et al., 2016) was developed recently for large-scale genomic selection projects, this technology allows hundreds to thousands of markers to be scored for less than US$ 5 per sample. CIMMYT in collaborations with Cornell University is testing how to implement genomic prediction on untested double haploid lines using rAmpSeq technology. Cost benefit analysis and genomic prediction accuracy results of this initiative will be reported in the near future.

## Conclusion

The main objectives of this study were to evaluate the *r*_*MG*_ value of the six trait-environment combinations in 22 bi-parental tropical maize populations genotyped with low-density SNPs and assess the effect of *h*^*2*^, TPS, and MD on *r*_*MG*_ estimation in bi-parental populations. Results of this study are clear, moderate *r*_*MG*_ means obtained for different trait-environment combinations, when 50% of the total genotypes was used as training population and ~200 SNPs were used for prediction. Architecture of predicted trait affects the *r*_*MG*_ value estimation, complex traits had lower *r*_*MG*_ means than those of less complex traits, and *r*_*MG*_ mean of the same predicted trait was higher in WW condition than in WS condition. For all the trait-environment combinations, *r*_*MG*_ value increased as the increase of *h*^*2*^, TPS, and MD. Both correlation and variance analyses showed that *h*^*2*^ is the most important factor on *r*_*MG*_ estimation. Among the three factors, *h*^*2*^ most significantly correlates with *r*_*MG*_ and explains the greatest percentage of the total variance of *r*_*MG*_ for almost all the target trait-environment combinations. Among the three factors, the MD was least associated with *r*_*MG*_ estimation for most of the trait-environment combinations, good *r*_*MG*_ values could be obtained in bi-parental maize populations, when the *h*^*2*^ of target trait is high and the genome is covered with sufficient markers (mean distance between markers is < 10–20 cM or around 150 markers evenly distributed the whole genome). Higher MD is required to achieve good *r*_*MG*_ values in bi-parental populations for complex traits with relative low *h*^*2*^ and strong genotype by environment interaction. Predictions between pairwise half-sib populations showed that the *r*_*MG*_ values for all the six trait-environment combinations were centered around zero, high-density and cost-effective genotyping platforms are required to apply genomic predictions across populations and implement GS routinely in the breeding programs. The trend observed in *r*_*MG*_ differed with the trend observed in *r*_*MG*_/*h* for the different trait-environment combinations, which indicated that both *r*_*MG*_ and *r*_*MG*_/*h* values should be presented in the GS studies to show the accuracy of GS and the relative accuracy of GS compared with phenotypic selection on various target predicted traits.

## Author contributions

XZ, BP, MO, FS, and YB designed, led, and coordinated the overall study. YB performed and coordinated the field experiments. KS performed and coordinated the genotyping work. AZ, HW, YL, SC, ZC, YR, JB, and JC carried out the analysis. XZ, AZ, HW, and HY wrote the manuscript.

### Conflict of interest statement

The authors declare that the research was conducted in the absence of any commercial or financial relationships that could be construed as a potential conflict of interest.
